# Interstitial Lung Disease in Systemic Lupus Erythematosus and Systemic Sclerosis: How Can We Manage the Challenge?

**DOI:** 10.3390/ijms24119388

**Published:** 2023-05-28

**Authors:** Patricia Richter, Anca Cardoneanu, Nicoleta Dima, Ioana Bratoiu, Ciprian Rezus, Alexandra Maria Burlui, Damiana Costin, Luana Andreea Macovei, Elena Rezus

**Affiliations:** 1 Department of Rheumatology, “Grigore T Popa” University of Medicine and Pharmacy, 700115 Iasi, Romania; 2 Clinical Rehabilitation Hospital, 700661 Iasi, Romania; 3 Department of Internal Medicine, “Grigore T Popa” University of Medicine and Pharmacy, 700115 Iasi, Romania; 4 “Sf. Spiridon” Clinical Emergency Hospital, 700111 Iasi, Romania

**Keywords:** systemic lupus erythematosus, systemic sclerosis, nintedanib, interstitial lung disease

## Abstract

Interstitial lung disease (ILD) is a severe and frequent manifestation of connective tissue diseases (CTD). Due to its debilitating potential, it requires serious evaluation and treatment. The prevalence of ILD in systemic lupus erythematosus (SLE) is still controversial. Therefore, in order to establish the diagnosis of ILD, an overlap syndrome must be excluded. Increasing the identification of SLE-associated ILD cases should become a target. To treat this complication, various therapies are now being proposed. To date, no placebo-controlled studies were conducted. Regarding another CTD, systemic sclerosis (SSc), SSc-associated ILD is considered one of the leading causes of mortality. The incidence of ILD varies among disease subtypes, being influenced by diagnostic method, but also by disease duration. Due to the high prevalence of this complication, all SSc patients should be investigated for ILD at the time of SSc diagnosis and during the course of the disease. Fortunately, progress was made in terms of treatment. Nintedanib, a tyrosine kinases inhibitor, showed promising results. It appeared to decrease the rate of progression of ILD compared to placebo. This review aimed to provide up-to-date findings related to SLE-associated ILD and SSc-associated ILD, in order to raise awareness of their diagnosis and management.

## 1. Introduction

Data from the literature on connective tissue disease-associated interstitial lung disease (CTD-ILD) focused on systemic sclerosis (SSc)-ILD and rarely on systemic lupus erythematosus (SLE)-ILD. SLE is a chronic, autoimmune condition that can present with a wide range of clinical and immunological manifestations. Pulmonary involvement in SLE is various. SLE-associated ILD, while rare, is a predictor of poor prognosis [1].

SSc is a heterogeneous autoimmune disorder characterized by multiorgan vascular and fibrotic abnormalities. On the other hand, pulmonary involvement in SSc is frequent, ILD being considered one of the most common cause of death. Despite being one of the most significant SSc complications, there is no established treatment for it yet. A new treatment for ILD is, therefore, definitely needed. Recently, nintedanib was successfully approved for SSc-ILD in Japan, Europe, and the United States.

## 2. Systemic Lupus Erythematosus

In addition to other systemic manifestations, pulmonary involvement has an impact on patients’ quality of life and disease prognosis [1]. ILD, a pulmonary manifestation of several connective tissue disorders (CTDs), frequently causes important morbidity and mortality [2]. Scleroderma is one of the CTD’s most frequently reported ILD; however, other CTDs, such as SLE, can also predispose to ILD. ILD can determine severe clinical symptoms, worsening survival rates in these patients [2]. The relationship between ILD and SLE is relatively rare [2,3,4,5,6]. However, individuals with overlapping connective tissue disease syndromes are highly prone to develop this manifestation [2]. Furthermore, an American Indian heritage was mentioned [7].

### 2.1. ILD in SLE

The most common lung conditions in SLE are pleuritis, pulmonary thromboembolism, diffuse alveolar hemorrhage and, less frequently, shrinking lung syndrome [2,8]. We may classify these various manifestations into: pleural (pleurisy or pleural effusion), lung parenchyma (interstitial lung disease or acute pneumonitis), as well as pulmonary vascular involvement (pulmonary hypertension, pulmonary embolism, or vasculitis). Moreover, we can find respiratory infections due to the immunosuppressive drugs used in SLE [9,10]. In some patients, lupus pneumonitis seems to be a precursor to chronic ILD [11]. When SLE-associated interstitial pneumonia (IP) was studied, the most common form was a chronic involvement, followed by a subacute and acute IP [12,13].

Although the mechanism of pulmonary involvement in SLE is unclear, the disease is known to have a high expression of type I interferon (IFN)-regulated genes, termed “IFN signature”. Additionally, type 1 IFNs are crucial for promoting autoantibody synthesis, neutrophil NETosis with subsequent dysregulation of immune tolerance in the lungs. Proinflammatory cytokines such as IFN-γ, tumor necrosis factor-α (TNF-α), interleukin-6 (IL-6), IL-8, IL-12 are present at higher levels in SLE patients and lung involvement [14].

Data on the pulmonary involvement in SLE are currently insufficient [15,16]. It is well known the essential role of cytokines in organ dysfunction in SLE. These patients have an increased serum, urine, and cerebrospinal fluid level of cytokines [15,16,17]. Increased serum levels of cytokines were found in SLE patients who associate pulmonary manifestations compared to cases without lung involvement [15,18].

Chemokines are essential in the pathogenesis of SLE associated-pulmonary fibrosis (PF). Some reports showed that SLE patients with PF were found to have higher levels of CX3C motif chemokine receptor 1 (CX3CR1) and CX3C chemokine ligand 1 (CX3CL1) [14,15]. Thus, Qiu et al. recently demonstrated that CX3CL1/CX3CR1 axis is linked to PF. Thus, this study raised the hypothesis that CX3CR1 may be a predictor of PF in SLE patients. Moreover, CX3CL1 and CX3CR1 interactions may become a promising target for the management of SLE-associated PF [15].

The identification of specific phenotypes in the serum of SLE patients who develop ILD and/on myositis was the objective of a recent study. Cotton et al. showed that KL-6, anti-Ro52, and anti-Ku were the most prevalent antibodies in patients with ILD/myositis [3].

It seems that ILD affects a small percentage of SLE patients, between 2 and 4% [2,8,19]. However, it is worth mentioning that, in some cohorts, a higher prevalence was found. A study from Japan described a prevalence of 29%. More interestingly, in many cases, the manifestation was present at the time of diagnosis of the autoimmune disease [2,20]. Others reported that pulmonary manifestations typically occur later in the course of the disease [21,22].

In terms of risk factors that may predispose to the development of ILD, age, and duration of autoimmune disease are both mentioned. One study found an average of 7.7 years between the onset of SLE and ILD [2,8]. Older age may also be related to the development of ILD [2,20]. An ILD prevalence of 30% was found in SLE patients over 50 years old [2,23]. A meta-analysis showed that late-onset SLE patients are more likely to develop pulmonary events than younger patients. A possible explanation would be that age may promote the immune senescence of the lung [21]. However, age was an independent risk factor for the onset of lung involvement in SLE patients in LUMINA cohort [21,24].

The association with other clinical manifestations such as Raynaud’s phenomenon or sclerodactyly in an overlap syndrome is considered to be the risk factor [9,11,25,26,27]. Chen et al enrolled 505 SLE patients and additionally observed the presence of tachypnea in 55 ILD patients [28]. Low levels of albumin and chest tightness/shortness of breath should indicate the presence of ILD, regarding a recent study that found statistical significant differences between the SLE-ILD and SLE-non ILD groups [29].

ILD can be divided into several manifestations: follicular bronchitis, organizing pneumonia (usual interstitial or lymphocytic interstitial pneumonia) and non-specific interstitial pneumonia (NSIP) [9,30,31,32,33]. The onset of the symptoms is usually insidious [34]. Regarding symptoms at presentation, patients may be asymptomatic or present cough and dyspnea [9,35]. Considering the asymptomatic presentation in most patients, this could draw attention to a subclinical course of the disease [5].

The characteristics of ILD include aberrant remodeling of lung tissue driven by an excessive synthesis and deposition of extracellular matrix as well as a defective repair of alveolar epithelial cell following chronic injury [15,36]. Vivero and Padera investigated different histological characteristics associated with ILD secondary to CTD. It seems that each CTD has a particular histological pattern. Using lung biopsy, a certain disease can be confirmed. In SLE-related NSIP, mononuclear or lymphoplasmacytic interstitial and peribronchiolar infiltrates were described [37].

Laboratory changes such as the presence of acute phase reactants in high titers, elevated double-stranded DNA (dsDNA) antibody levels, and a low level of complement should indicate a possible pulmonary involvement related to SLE [9]. Other antibodies such as anti-La, anti-Scl-70 and anti-U1RNP predispose to the development of ILD [9,37]. Vasser et al. confirmed the increased prevalence of auto-antibodies; for example, anti-La, anti-Sm, RNP, and lupus anticoagulant in their cohort [7]. Interestingly, Chen et al. found remarkably lower levels of anti-dsDNA antibodies and elevated levels of serum complement C3 in a cohort of patients with SLE-associated ILD [28].

In order to establish the diagnosis of SLE-associated ILD, an overlap syndrome should be firstly excluded. This possible cause can lead to a false diagnosis. Then, a high resolution-computed tomography (HR-CT) should be performed [9,38]. SLE-ILD is often clinically diagnosed, using frequently imaging methods such as HRCT and excluding other possible causes [39]. It is very important to make a correct differential diagnosis with: infections, tuberculosis, pulmonary oedema, cancers or even sarcoidosis [34,40].

Additional functional tests may reveal asymptomatic abnormalities, usually a restrictive dysfunction and a decrease in diffusing capacity for carbon monoxide (DLCO) [9,41].

At the onset of the disease, the radiological image may be normal. Bibasilar irregular linear opacities may also be found. Subsequently, it can be highlighted a diffuse or bibasilar infiltrates, pleural disease, or a honeycombing aspect [34]. Ground glass opacities were reported having a great frequency [19], followed by pulmonary consolidation, honeycombing aspect, or even traction bronchiectasis [42,43]. The identification on thoracic CT of two novel distinct fibrosis patterns (the island-like fibrosis and heterogeneous lung destruction signs) may be considered a starting point in differentiating patients with idiopathic pulmonary fibrosis from those with CTD-related ILD [44].

HR-CT is used to identify lung involvement and the specific pattern of disease [34]. Some studies reported NSIP as the most frequent pattern in SLE patients with ILD [45,46], although data are still controversial.

### 2.2. Management of ILD in SLE

Information on treatment in ILD-SLE is limited [2]. Some data from the literature are presented in Table 1. All patients with SLE should use hydroxychloroquine as their first-line treatment, according to the European League Against Rheumatism (EULAR). Glucocorticoids may also be added, if necessary. Immunosuppressive agents are indicated in refractory cases or even as the initial therapy in life-threatening cases [47].

Current treatments only reduce the progression of the disease [48]. The choice of a certain immunosuppressive treatment is based on the experience in scleroderma-associated ILD [49]. No placebo-controlled trials were performed in order to assess the efficacy and safety profile of corticosteroids and immunosuppressant drugs for the treatment of ILD secondary to SLE [34].

The therapeutic strategy is based on expert opinion [50]. It was recently agreed that corticosteroids should be used as first-line therapy associated with cyclophosphamide (CYC) or mycophenolate mofetil (MMF), followed by either rituximab or intravenous immunoglobulins (IVIG) [2,51]. More precisely, Koo et al. indicated, as first-line treatment for mild and moderate forms, the use of corticosteroid and azathioprine/MMF, drugs used also as a maintenance therapy; for refractory forms, high-dose steroids and a steroid-sparing agent (for example, CYC) can be used [4,50,51,52]. In most patients with SLE-ILD, systemic corticosteroids may normalize inspiratory vital capacity and DLCO, despite there being minimal evidence regarding their efficacy [53].

Currently, the impact of biological therapies on patients having various forms of ILD is being investigated [48]. Belimumab, a recombinant monoclonal antibody that targets the B-cell activating factor (BLys), is the first biological agent approved in SLE treatment [54]. Rituximab may be useful as second-line therapy [50,55]. Notably, patients having a severe and progressive disease may respond to rituximab, which may also prevent lung transplantation [56]. Intravenous immunoglobulin (IVIg) and plasmapheresis are used to treat refractory cases [39]. Antifibrotic treatments and lung transplantation should be properly considered for patients with fibrotic CTD-associated ILD and a progressive and severe disease course [2].

**Table 1:** Treatment of SLE-associated ILD.

Author	Year	Patients	Endpoint	Clinical Results
Weinrib et al. [53]	1990	14 SLE patients with ILD	Evaluation of the clinical course of ILD in SLE patients after CS therapy	High doses of oral prednisolone showed significant improvement in 3 patients.
				Improvement of respiratory symptoms in 6 patients when systemic steroids were used
Eiser et Shanies [57]	1994	2 SLE patients with ILD	The efficacy of the early use of IPC for SLE-associated ILD	VC showed significant improvement after intravenous CYC treatment
Fink et al. [58]	1995	1 SLE patient with ILD	The outcomes of methotrexate treatment in one case of SLE-associated ILD	Notable improvement in lung function after oral methotrexate
Swigris et al. [59]	2006	28 CTD patients with ILD	Safety and tolerability of MMF in CTD-ILD patients and its impact on lung function	MMF was highly safe and well tolerated in this cohort
Lim et al. [60]	2006	1 SLE patient with ILD	The evolution of the first patient with SLE-pneumonitis refractory to conventional medication treated with rituximab	Improvement in clinical parameters, disease activity and spirometry values post rituximab therapy
Okada et al. [61]	2007	17 CVD patients with ILD	The efficacy and safety of the administration of CYC infusion for CVD-associated ILD	CYC pulse therapy ameliorated pulmonary symptoms in all patients;
				Beneficial effects also on VC and DLCO
Sumida et al. [62]	2014	1 Overlap case (SLE and SSc) with ILD	The follow-up at baseline and 6 months later after two cycles of rituximab	Treatment with rituximab showed an improvement in respiratory symptoms, reduction in FVC and DLCO functional tests;
				Improvement in SLE activity
Yang et al. [63]	2017	1 SLE patient with ILD	The effect of an oral antifibrotic agent -pirfenidone combined with corticosteroids	One year treatment with pirfenidone and corticosteroids showed a positive effect on malar rash, respiratory symptoms, DLCO, FCV and HRCT aspect
Nawata et al. [64]	2020	1 SLE patient with IP	The efficacy of MMF in a SLE complicated by PAH/IP	Improvement of SLE activity score and PAH/IP following MMF treatment
Robles-Perez et al. [56]	2020	18 CTD-related pulmonary disease	The effect of rituximab in CTD-associated lung conditions patients who were eligible for a lung transplant	Following a two-year rituximab treatment, DLCO considerably improved
Mwagni et al. [54]	2021	1 Overlap syndrome (SLE and Scleroderma) with ILD	The effect of belimumab	Total lung capacity, chest CT opacities improved, right ventricular systolic pressure normalized during two years treatment with belimumab
Jordan et al. [39]	2021	1 SLE patient with ILD	The evolution on a SLE associated-ILD patient treated with Intravenous Immunoglobulin	Successful response at Intravenous Immunoglobulin

IP = interstitial pneumonia; PAH = pulmonary hypertension; MMF = mycophenolate mofetil; SSc = systemic sclerosis; FVC = reduced forced vital capacity; DLCO = diffusing capacity for carbon monoxide; LP = lupus pneumonitis; CVD = collagen vascular diseases; CYC = cyclophosphamide; VC = vital capacity; CTD = connective tissue diseases; HR-CT = high resolution CT; IPC = intravenous pulse cyclophosphamide.

In SLE, it is mentioned that the clinical evolution of ILD is gradual and frequently stabilizes over time [9,53]. In terms of prognosis, it is not yet known whether ILD can be considered a negative factor in SLE patients. One study claimed that ILD is an independent risk factor for mortality. In another cohort, no significant differences in survival were found in patients with concomitant ILD [2,8,20].

## 3. Systemic Sclerosis

### 3.1. ILD in SSc

Pulmonary involvement in SSc is frequent and ILD is considered one of the most common causes of death having a 10-year mortality up to 40% [65]. The incidence of ILD varies significantly between disease subtypes, but it is also influenced by the diagnostic method used or by disease duration [66]. SSc-ILD is most frequently defined by NSIP in up to 78% of the cases [67].

There are certain clinical characteristics that were associated with ILD presence. Patients with lcSSc (limited systemic sclerosis) and positive anticentromere antibodies (ACA) are less likely to present ILD compared to those having a dcSSc (diffuse systemic sclerosis), anti-Scl-70, and anti-topoisomerase I antibodies (ATA) [66]. Additionally, male gender, higher levels of mRSS (modified Rodnan Skin Score), the presence of gastroesophageal reflux disease, digital ulcers, and pulmonary hypertension were associated with the presence of ILD [68]. Additionally, the degree of skin involvement assessed by mRSS is considered to be a prognostic factor for progressive ILD, characterized by a FVC-predicted decline of more than 10% over a 12-month period [69]. ATA is also a prognostic factor for a decreased pulmonary function and severe ILD in patients with short disease duration [66,70].

ILD linked to SSc has a complicated and poorly known pathogenesis which includes fibrosis and various degrees of inflammation. As a consequence of innate and adaptive immune system inaccurate activation, fibroblasts are activated and myofibroblasts produce excessive extracellular matrix [65,66]. There are three main stages that lead to the development of ILD in SSc. In the early phase, there is a triggering event that occurs in a susceptible subject determined by genetic and epigenetic factors. Early inflammation leads to a pronounced pro-fibrotic phenotype fibroblasts and TGF-β activation. In the established phase the ongoing inflammation and failure of resolution processes determine an excessive matrix deposition. From this point forward, some participants will advance to stability and even regression and some will progress to a more severe form of ILD [67].

All SSc patients need to be screened for ILD at the time of SSc diagnosis and over the disease course due to the high prevalence of this complication. Part of the patients may describe typical symptoms such as dyspnea, non-productive cough, or fatigue. However, in some cases, there are no specific symptoms and, in these situations, screening for ILD is extremely important for an early diagnosis [71]. Screening and diagnosis are made with the following elements: clinical examination, pulmonary function testing (including forced vital capacity [FVC] and DLCO), HRCT, and chest X-rays [71,72]. However, the gold standard for diagnosis is HRCT, as it is more sensitive than X-ray [73].

### 3.2. Management of ILD in SSc

Although ILD is one of the most important complications in SSc, a definite treatment is not yet standardized. It is based mostly on an immunosuppressive strategy and a “wait and watch” approach [74,75]. Methotrexate, cyclophosphamide (CYC), or mycophenolate mofetil (MMF) can be used, although they do not have a very high efficacy and are associated with important adverse effects. Thus, a new treatment for ILD is highly needed; since 2019, various studies on rituximab, tocilizumab, and nintedanib were published. Following study findings, tocilizumab was authorized for SSc-ILD in March 2021 in United States and rituximab was approved in Japan in September 2021. Recently, nintedanib was also successfully approved for SSc-ILD in the United States, Europe, and Japan [74,76].

Nintedanib is a tyrosine kinases inhibitor previously approved for idiopathic pulmonary fibrosis following INPULSIS studies [77]. Its effect on pulmonary fibrosis was observed in both in vitro and in vivo studies, reducing bleomycin or silica pulmonary fibrosis, having a significant antifibrotic and anti-inflammatory action [78]. Moreover, the in vitro effect persisted in circulating fibrocytes from SSc patients, preventing their transition into myofibroblasts [79].

After nintedanib proved antifibrotic effects in patients with idiopathic pulmonary fibrosis, it was further investigated to see if the beneficial effects can be demonstrated in a fibrosis. Thus, the safety and efficacy of nintedanib was investigated in SENSCIS trial, conducted between 2015 and 2017. It was a randomized, double-blind, placebo-controlled trial that involved 576 patients with SSc; the patients were over 18 years old, with a history of Raynaud’s phenomenon of at least 7 years prior to screening, from 32 countries. Patients were randomized 1:1 into a placebo group and a treated group receiving 150 mg of nintedanib twice daily. The extent of pulmonary fibrosis was diagnosed using HRCT with a mean extent of fibrosis of 36 ± 21.3%. In almost half of the participants, MMF was administrated at baseline. After 52 weeks, 232 patients from the nintedanib group and 257 from the placebo group completed the study. In the nintedanib group, the adjusted yearly change in FVC was 52.4 mL/year, while in the placebo group, it was 93.3 mL/year, proving a significantly suppressed reduction in FVC decline. Furthermore, in patients receiving MMF, the mean adjusted annual decrease in FVC was −40.2 mL in the nintedanib group compared to −66.5 mL in the placebo group. Either way, nintedanib proved its beneficial effects compared to placebo, and a more significant effect when combined with MMF [80]. Further on, many scientists performed extensive studies using the same cohort of patients from SENSCIS trial and demonstrated various results presented in Table 2.

## 4. Comparison between ILD SLE and SSc

As shown in Table 3, the prevalence of ILD in SLE differs from SSc. ILD is a rare manifestation in SLE, whereas in SSc, it occurs frequently up to 75%. The HLA genetic background was described in ILD-associated SSc, whereas in SLE, it is poorly understood. Several antibodies were described in relation to CTD, with some antibodies occurring in both diseases. NSIP is the most common manifestation of ILD in both SSC and SLE. It appears that pulmonary manifestation occurs a few years after CTD diagnosis. The clinical manifestation is also similar in these two diseases. It can range from asymptomatic to common pulmonary manifestations such as dyspnea or cough. For diagnosis and staging, both diseases require HT-CT. Treatment primarily involves the treatment of the underlying disease. In SLE, various therapies were tried, starting from corticosteroids to biological treatment or antifibrotic agents [9,65,95].

## 5. Conclusions

Many organs and systems can be affected in SLE and SSc. Although the major focus is on manifestations that lead to high mortality, lung damage is an important predictor of disease progression and prognosis. This article presented a comprehensive review of the current literature data on the management of ILD in SSc and SLE and information on the use of nintedanib in SSc. It gained significant attention in recent years for its potential therapeutic benefits.

Even if pulmonary fibrosis is a rare manifestation in SLE, prompt diagnosis of this complication is truly important. There is still ongoing controversy about the true prevalence of ILD in SLE, given the overlapping syndromes. Overall, the prognosis for patients with SLE-ILD is encouraging.

On the other hand, in SSc, ILD is a frequent manifestation associated with high morbidity and mortality. The prevalence is highly variable given the different diagnostic method used. There are limited data on the management and treatment of this complication, but new therapies seem promising, and nintedanib emerged as a treatment option for patients with ILD in SSc, improving lung function and slowing the progression of ILD. Despite the fact that it has a favorable safety profile, the optimal dosing and treatment duration remain to be evaluated through further studies.

In conclusion, nintedanib represents a significant advancement in the field of fibrotic diseases, and its integration into clinical practice has the potential to improve patient outcomes and quality of life in SS. On the other hand, in SLE cases, immunosuppressive treatment remains a valid option.

## Figures and Tables

**Table 2:** Extension studies that used the patients from SENSCIS trial.

Author	Year	Patients	Endpoint	Clinical Results
Flaherty et al. [81]	2019	The INBUILD study included 663 patients of which 39 had SSc (23 received nintedanib vs. 16 placebo)	Primary: annual decline in FVC (mL/year) after 52 weeks of treatment	The annual decline rate of FVC was significantly lower in the nintedanib group in patients with UIP-like fibrotic pattern.
			Secondary: change in the total score of K-BLID questionnaire and the time until the first acute exacerbation of ILD or death within 52 weeks.	
Kuwana et al. [82]	2020	The study included Japanese patients (*n* = 70) from SENSCIS trial:	Primary: annual decline in FVC (mL/year) after 52 weeks of treatment	17.6% of the nintedanib group and 5.6% of the placebo group discontinued the treatment due to adverse effects.
		36 patients received placebo vs. 34 nintedanib.	Secondary: changes from baseline in mRSS and SGRQ scores after 52 weeks.	The decline in FVC from baseline was smaller for patients in the nintedanib group compared to placebo at 52 weeks and the effect persisted up to 100 weeks, similar between Japanese and non-Japanese participants.
				The mRSS and SGRQ scores and the rate of adverse effects were similar between Japanese and non-Japanese patients.
Seibold et al. [83]	2020	The study included the same patients (*n* = 576) from the SENSCIS trial.	Primary: data about possible digestive (diarrhea, liver enzyme, and bilirubin elevation) and cardiovascular adverse effects.	The most common adverse effect was diarrhea in both groups (75.7% in the nintedanib group and 31.6% in the placebo group).
				Almost half of these patients had the first episode in the first 30 days of treatment.
				70.2% of them had one or two diarrhea episodes 94% of the cases being described as mild or moderate.
		288 received placebo and 288 nintedanib.	Secondary: the predisposition to develop intestinal side effect.	Antidiarrheal medication was prescribed in 48% of nintedanib patients and in 9% of placebo group.
				Dose reduction was considered in order to maintain the continuity of the treatment in 40.6% of the nintedanib group participants.
				Other digestive adverse events (nausea, vomiting, abdominal pain) or weight loss were more common in patients treated with nintedanib.
Azuma et al. [84]	2021	The study included Asian patients (*n* = 143) from the SENSCIS trial.	Primary: the annual decline in FVC rate (mL/year), the baseline changes in mRSS and SGRQ scores after 52 weeks	Fewer Asian participants received MMF and methotrexate compared to non-Asian patients.
		62 were from nintedanib group.	Secondary: adverse events developed over 52 weeks of treatment.	The benefit of slowing the FVC decline and the adverse events described were similar between Asian and non-Asian patients throughout the entire study.
Highland et al. [85]	2021	The study included the patients from the SENSCIS trial (*n* = 576).	Primary: the annual decline in FVC (mL/year) after 52 weeks of treatment	More than 90% of cases continued MMF treatment until week 52.
		They were divided based on the treatment with MMF at baseline (median dose of 2000 mg): 139 from nintedanib group and 140 from placebo group.	Secondary: changes from baseline in mRSS and SGRQ scores after 52 weeks. The annual percentage rate of FVC decline, the absolute change in mL of FVC baseline the absolute and relative decrease in FVC of more than 5% predicted and more than 10% predicted were also evaluated.	Nintedanib usage was associated with a reduced rate of decline in FVC regardless of the association with MMF
				MMF association did not improve skin fibrosis or quality of life in both nintedanib and placebo groups and had a similar adverse event profile.
Maher et al. [86]	2021	The study included the same patients (*n* = 576) from the SENSCIS trial.	Primary: the absolute decrease/increase in FVC	A decline in FVC% predicted was described in 55.7% of participants in nintedanib group and in 66.3% of participants in placebo group.
				After 52 weeks, 13.6% of nintedanib group had an absolute decline in FVC between 5–10% predicted, while only 3.5% had an absolute decline in FVC between 10 and 15% predicted. In placebo group, the absolute decline in FVC and death were present in a higher proportion.
			Secondary: The MCID for improvement, worsening or stability in FVC.	The absolute decrease in FVC at 52 weeks of ≥3.3% (the proposed MCID) was 34.5% in nintedanib group versus 43.8% in placebo group.
				The absolute increase in FVC at 52 weeks of ≥3.3%(the proposed MCID) was 23% versus 14.9% in nintedanib vs. placebo groups.
Allanore et al. [87]	2022	The study included patients from the SENSCIS-ON trial who completed the 52 weeks of the SENSCIS trial.	Primary: reported adverse events from the first day of treatment until 52 weeks or to the last drug intake plus 7 days for those who prematurely discontinued treatment.	Diarrhea was reported in 68% of the cases who continued nintedanib and in 68.8% in the group of patients who initiated nintedanib as the most frequent adverse effect, having a mild or moderate intensity.
		It included *n* = 444 patients, 197 who continued nintedanib and other 247 who initiated the treatment.	Secondary: absolute increase and decrease in FVC, changes in mRSS, SGRQ total score, UCLA SCTC GIT 2.0 score.	Serious adverse events were documented in approximately 20% of the cases from both groups, the most important being pneumonia.
		Almost half of the patients from both groups were taking MMF.		The frequency of adverse events did not differ in SENSCIS-ON as compared to SENSCIS trial.
				Fewer patients discontinued the treatment in SENSCIS-ON group.
				The progression of FVC over 52 weeks was similar.
Assassi et al. [88]	2022	The study included the same patients (*n* = 576) from SENSCIS trial.	Primary: the annual decline in FVC (mL/year) after 100 weeks	In both intent-to-treat and on-treatment analysis, the adjusted mean annual rate of decline in FVC after 100 weeks was slower in patients with nintedanib compared to placebo.
			Secondary: the effect of nintedanib in patients who followed the treatment until the end of the study	
Denton et al. [89]	2022	The study included the same patients (*n* = 576) from SENSCIS trial.	Primary: the relationship between the degree of fibrotic ILD and the absolute rate of FVC decline in 52 weeks.	The degree of fibrotic ILD on HRCT at baseline and the decline in FVC predicted at 52 weeks were not related with the overall population or with subgroups of participants.
				the extent of fibrotic ILD at baseline was not associated with the rate of decline in FVC over 52 weeks.
			Secondary: the extent of fibrotic ILD and predicted FVC at baseline	the higher the FVC predicted values at baseline the greater the decline in FVC during the trial, suggesting that a greater respiratory reserve leads to a higher decline in FVC.
				nintedanib is beneficial irrespective of the fibrotic ILD extent on HRCT.
Kreuter et al. [90]	2022	The study included the same patients (*n* = 576) from the SENSCIS trial.	Primary: annual decline in FVC (mL/year) over 52 weeks.	Baseline PRO measures scores were worse in dcSSc and correlated with a lower FVC, greater extent of fibrosis on HRCT, higher mRSS, presence of GERD, and other upper gastrointestinal symptoms in patients receiving MMF. Additionally, it was linked with hospitalization during the study and with supplemental oxygen usage.
			Secondary: changes from baseline in PRO measures questionnaires: SGRQ, FACIT-dyspnea, and HAQ-DI after 52 weeks.	After 52 weeks, in patients with FVC ≥ 70% predicted, PRO measures scores had better results compared with FVC ˂ 70% predicted.
Kreuter et al. [91]	2022	The study included the same patients (*n* = 576) from the SENSCIS trial.	FVC decline rate in patients who were hospitalized for an all-cause event or SSc-related causes.	After 52 weeks, 13.7% of the participants experienced an all-cause hospitalization event or died, of which 7.4% were related to SSc.
				FVC decline and time to first all-cause hospitalization or death were linked with statistically significant results after 52 weeks.
		78 were hospitalized for all-cause events, 42 for SSc causes and 75 were admitted in ER or hospital followed by admission to ICU.	The risk of first hospitalization events related to FVC decline rate.	FVC decline was also correlated with time to first SSc-related hospitalization or death, yet no relationship was found between FVC decline and risk of hospitalization or death of any cause.
				The reduction in FVC decline resulting after nintedanib treatment may reduce the risk of hospitalization.
Kuwana et al. [92]	2022	The study included the same patients (*n* = 576) from the SENSCIS trial divided after ATA status, baseline MRSS and SSc subtype.	Primary: the annual decline in FVC over 52 weeks in the 3 groups.	Almost 60% of the patients were ATA-positive in both groups. Patients in the placebo group who were ATA-positive or negative had the same adjusted yearly rate of reduction in FVC.
		In the nintedanib group there were 173 ATA positive patients versus 177 in the placebo group.		Compared to ATA-positive patients, nintedanib’s effect on lowering the annual rate of decline in FVC was more pronounced in the ATA-negative patients.
		There were 218 patients with mRSS < 18 in the nintedanib group and 226 in the placebo group.	Secondary: the correlations between FVC and MRSS at baseline versus week 52 and the decline rate of FVC considering MRSS at baseline a continuous variable.	The reduction in mRSS was similar between nintedanib and placebo groups regardless of the ATA status.
				In the placebo group, the rate of decline in FVC over 52 weeks was similar in ATA positive and negative patients, higher in patients with a mRSS ≥ 18 at baseline compared to those with a mRSS ˂ 18 and higher in patients with dcSSc compared to lcSSc. Nintedanib had no effect on reducing the mRSS in any of the subgroups.
Maher et al. [93]	2022	The study included the same patients (*n* = 576) from the SENSCIS trial.	Primary: the decline in FVC at baseline and over 52 weeks in the two groups	Participants from SENSCIS trial had a substantial impairment in FVC at baseline, with an FVC (mL) over 25% (around 950 mL) lower than it would be anticipated in those without lung disease. As the average duration of SSc in the SENSCIS study was about 3.4 years, the predicted loss of lung function was about 280 mL/year since the onset of SSc.
		They were compared to a matched population for age, sex, ethnicity and height.	Secondary: to estimate the „effective lung age” and compare the results to the real age	After 52 weeks, the decline in FVC was four-fold greater in the placebo group, while in the nintedanib group it was only two-fold greater compared to hypothetical healthy references.
Volkmann et al. [94]	2022	The study included 574 patients from the SENSCIS trial with information on cough and dyspnea. 353 had both cough and dyspnea, 156 had only cough or dyspnea, 65 no complaints.	Primary: the annual decline of FVC over 52 weeks; the proportion of patients with relative/absolute decline in FVC > 5% and >10% at week 52; time to absolute decline in FVC >10% or death over 52 weeks in all the subgroups based on cough and/or dyspnea.	Most of cases reported coughing (76.6%) several days a week and some (23.4%) reported coughing a few days a month.
		Cough was described in 229 patients from the nintedanib group and in 232 of the placebo group.	Secondary: changes in SGRQ scores at week 52.	In the dyspnea group, 70% reported it several days a week and 29.6% described dyspnea a few days a month.
		Dyspnea was present in 209 of the nintedanib participants and 193 of the placebo participants.		Both cough and dyspnea were associated with a greater extent of fibrotic ILD, a lower FVC % predicted and a lower DLCO% predicted at baseline.
				The rate of FVC decline after 52 weeks was similar in placebo patients regardless of the presence of cough or dyspnea.
				In the nintedanib group, the effect of reducing FVC decline was more pronounced in those without cough or dyspnea, although no heterogeneity was observed between the subgroups.

ATA—anti-topoisomerase antibodies; ER—emergency room; FACIT—Functional Assessment of Chronic Illness Therapy; FVC—forced vital capacity; HAQ-DI—Health Assessment Questionnaire Disability Index; HRCT—high resolution computed tomography; ICU—intensive care unit; ILD—interstitial lung disease; K-BLID—King’s Brief Interstitial Lung Disease questionnaire; MCID—minimal clinically important differences; mRSS—modified Rodnan skin score; *n*—number of patients; PRO—patient-reported outcome; SGRQ—St. George respiratory questionnaire; UCLA SCTC GIT 2.0—University of California Los Angeles Scleroderma Clinical Trial Consortium Gastrointestinal tract v2.0.

**Table 3:** Comparison between ILD in SLE and SSc.

	SLE	SSc
Frequency	2–4%	75%
Risk factors	Older age Longstanding disease	dcSSc male sex African American race Nailfold capillary abnormalities Digital ulcers Longer disease duration Pulmonary hypertension
Genetics	-	HLA-DRB1*11 HLA-DRB1*301
Antibodies	Anti-dsDNA Anti-La Anti-Scl-70 Anti-U1RNP Anti-Sm lupus anticoagulant	Anti-topoisomerase I Anti-neutrophil cytoplasmic antibody Anticardiolipin Anti-Ro52 Anti-NOR90 Anti-U11/U12 Anti-Th/To Anti-polymyositis-scleroderma
Type	NSIP organizing pneumonia follicular bronchitis	NSIP UIP PPFE
Onset	Within an average of 7.7 years of SLE onset	Within 5 years of the first non-Raynaud phenomenon symptom.
Pathology	An aberrant inflammatory response due to cytokine release; The impaired apoptosis and abnormal fibroblast proliferation leading to alveolar injury was described.	An injury to the alveolar epithelium, the vasculature, or both have been proposed as the initial event, followed by an aberrant immune response with fibroblast recruitment and activation. Extracellular matrix overproduction and an important scarring process replace the standard pulmonary architecture. The fibrotic variant of NSIP is more frequent than the cellular variant. Survival is not different between the two types of NSIP variants.
Clinical	May be asymptomatic Dyspnoea Tachypnea Cough Possible evidence of scleroderma	Dyspnoea Non-productive cough fatigue Velcro-like crackles on auscultation
Treatment	Hydroxycholoquine Glucocorticoids CYC MMF Belimumab/Rituximab IVIg Antifibrotic treatments Lung transplantation	CYC MMF Rituximab Nintedanib Bone marrow or lung transplantantion
Diagnosis	HRCT Pulmonary function tests	HRCT Pulmonary function tests

dcSSc—diffuse systemic sclerosis; lcSSc—limited systemic sclerosis; NSIP—non-specific interstitial pneumonia; CYC—cyclophosphamide; MMF—mycophenolate mofetil; IVIg—intravenous immunoglobulin; HR-CT—high resolution computed tomography; UIP—usual interstitial pneumonia; PPFE—pleuroparenchymal fibrosis.

## Data Availability

Not applicable.
